# New records in vascular plants alien to Kyrgyzstan

**DOI:** 10.3897/BDJ.2.e1018

**Published:** 2014-01-21

**Authors:** Georgy Lazkov, Alexander Sennikov

**Affiliations:** † Institute of Biology and Soil Science, Bishkek, Kyrgyzstan; ‡ University of Helsinki, Helsinki, Finland

**Keywords:** Casual aliens, Central Asia, ephemerophytes, established aliens, naturalization, range expansion, secondary distribution area

## Abstract

A series of brief notes on distribution of vascular plants alien to Kyrgyzstan is presented. A further expansion of *Anthemis ruthenica* (Asteraceae), *Crambe orientalis* (Brassicaceae) and *Salvia aethiopis* (Lamiaceae) in northern and northwestern Kyrgyzstan is recorded. The first record of *Chenopodium vulvaria* (Amaranthaceae) from the northern side of Kyrgyz Range is confirmed, and the species was found for the second time in Alay Range. The ephemerous occurrence of *Hirschfeldia incana* (Brassicaceae) in Central Asia is recorded for the first time from Fergana Range. *Tragus racemosus* (Poaceae) is first recorded from the Chüy Depression as an ephemerous alien. *Arrhenatherum elatius*, escaped from cultivation and locally established, is new to the country. The second record of established occurrence of *Centaurea solstitialis* (Asteraceae) and an ephemerous occurrence of *Glaucium corniculatum* (Papaveraceae) are presented. Complete information is collected about the occurrence of every mentioned species in Kyrgyzstan.

## Introduction

The first checklist of the flora of vascular plants of Kyrgyzstan (Lazkov and Sultanova 2011) listed 3869 species of which 71 species (1.8%) were considered introduced aliens. So far very little information is available about alien plants in the country, mostly in the form of unannotated records scattered in “grey” literature. In the absence of special studies on alien plants and their populations, no comprehensive summary about distribution of alien plants in Kyrgyzstan has been published, and the status of many published records (established vs. casual) has not been ascertained yet.

The present collection of new records provides available information on 9 species of vascular plants, either first reported from the country or with poorly known distributions there. The majority of new records comes from the field season of 2013. In every case we did our best to trace previously published or unpublished herbarium records which are assembled altogether, taxonomically verified and mapped here. The flora of Kyrgyzstan is still very imperfectly studied, and the present contribution aims at completing the gaps in the only checklist in existence to date (Lazkov and Sultanova 2011).

## Materials and methods

Records of alien plants from Kyrgyzstan obtained by the authors during the field season of 2013 were checked for novelties against the information published in *Flora of the Kirghiz SSR*, vols. 1–11 (1952–1965) and Suppl. 1–2 (1967–1970); *Conspectus florae Asiae Mediae* = *Manual of Vascular Plants of Central Asia*, vols. 1–10 (1968–1993); Checklist of vascular plants of Kyrgyzstan (Lazkov and Sultanova 2011); and various taxonomic treatments and accounts of plant distributions. The collections of the Institute of Biology and Soil Science, Kyrgyz Academy of Sciences, Bishkek (FRU), the authors’ collections and older field observations were screened for records of alien plants in order to complement our new data. The records were mapped using a GPS navigator with WGS84 datum (specimens collected by A.S. & G.L., recorded positions) and Russian printed maps with Pulkovo-1942 datum (other collections and observations, estimated positions).

Species (their populations or colonies) were treated as alien to the territory when they were thought to have arrived by means of human intervention, intentional or not, disregarding the distance of transportation, and to occur without past or present targeted assistance of man (cf. Pyšek et al. 2012). The assessment of invasion status (casual, naturalized, or invasive) follows Richardson et al. 2000. Details on the habitats and populations (colonies) of species are provided when known; however, the data are usually scanty because of the incidental character of records.

The BGN (United States Board on Geographic Names) / PCGN (Permanent Committee on Geographical Names for British Official Use) romanization of the Kyrgyz and Kazakh language is employed to transliterate collection labels originally in Cyrillic. The romanization of toponyms in Kyrgyzstan is based on the official standard of the Cyrillic spelling (Ömürzakov et al. 1988). The toponyms expressed by composite words are hyphenized by tradition. Delimitation of mountain ranges and depressions is given according to Ömürzakov et al. 1988.

## Data resources

Specimen information is deposited in the database of records in vascular plants of Kyrgyzstan (Sennikov and Lazkov 2012) that is also published through the Global Biodiversity Information Facility (GBIF).

## Taxon treatments

### Chenopodium vulvaria

L. 1753

urn:lsid:ipni.org:names:165311-1

#### Materials

- **Type status:** Other material. **Occurrence:** catalogNumber: 23; recordedBy: G.Lazkov; **Taxon:** family: Amaranthaceae; genus: Chenopodium; specificEpithet: vulvaria; taxonRank: species; scientificNameAuthorship: L.; **Location:** continent: Asia; country: Kyrgyzstan; stateProvince: Chüy Region; locality: E of Kara-Balta Town, factory area (under construction); decimalLatitude: 42.798333; decimalLongitude: 73.888611; **Identification:** identifiedBy: G.A.Lazkov; dateIdentified: 22/06/2013; **Event:** eventDate: 22/06/2013; year: 2013; month: 6; day: 22; habitat: ruderal places; eventRemarks: alien plant; **Record Level:** collectionID: 88420; institutionCode: FRU; basisOfRecord: specimen
- **Type status:** Other material. **Occurrence:** catalogNumber: 24; recordedBy: G.Lazkov; **Taxon:** family: Amaranthaceae; genus: Chenopodium; specificEpithet: vulvaria; taxonRank: species; scientificNameAuthorship: L.; **Location:** continent: Asia; country: Kyrgyzstan; stateProvince: Osh Region; locality: Alay Range: Nookat, slopes; verbatimElevation: 2000; decimalLatitude: 40.235; decimalLongitude: 72.558; **Identification:** identifiedBy: G.A.Lazkov; **Event:** eventRemarks: alien plant; **Record Level:** basisOfRecord: observation
- **Type status:** Other material. **Occurrence:** catalogNumber: 25; recordedBy: I.Gubanov; **Taxon:** family: Amaranthaceae; genus: Chenopodium; specificEpithet: vulvaria; taxonRank: species; scientificNameAuthorship: L.; **Location:** continent: Asia; country: Kyrgyzstan; stateProvince: Chüy Region; locality: Kara-Balta ravine; decimalLatitude: 42.798333; decimalLongitude: 73.888611; **Identification:** identifiedBy: I.A.Gubanov; dateIdentified: 08/14/1961; **Event:** eventDate: 08/14/1961; year: 1961; month: 8; day: 14; eventRemarks: alien plant; **Record Level:** collectionID: 15550; institutionCode: MW; basisOfRecord: specimen

#### Native distribution and occurrence in Central Asia

Pratov 1972 uncritically assumed *Chenopodium vulvaria* to occur throughout Central Asia, probably as native to the whole area because he included no mention on its status. However, Mosyakin 1996 analysed its distribution and concluded that this species is native to the Mediterranean and South-West Asia, being alien elsewhere. *Chenopodium vulvaria* is certainly native to the *Flora Iranica* area (Uotila 1997; Uotila, pers. comm.) but is considered exclusively ruderal and synanthropic in Tajikistan (Sidorenko et al. 1968) and Uzbekistan (Botschantzev 1953). Its distribution in eastern Kazakhstan (Goloskokov 1960) and southern Siberia (Lomonosova 1992) is limited, fragmented, and also connected with human activities.

#### Occurrence in Kyrgyzstan

Nikitina 1955 was first to report this rare synanthropic species as ruderal in one place in Alay Range. Later she (Nikitina 1967) dismissed the record as erroneous. Indeed, no relevant specimens have been uncovered in the collections of FRU which are the basis of Nikitina’s treatments.

The first confirmed record of this species in Kyrgyzstan, documented by a specimen, was made in 1961 in the ravine of Kara-Balta river (Gubanov 1970), presumably close to its entrance, on the N side of Kyrgyz Range. This ravine is facing to Kara-Balta Town situated in the Chüy Depression; the southern part of the town (close to the ravine) is a vast industrial area since the Soviet times, including a large ore grinding factory which used imported ores.

This territory was revisited in 2013 in order to explore the area of the oil refinery factory (then under construction) east of Kara-Balta Town. The construction activities brought a number of alien plants, and the presence of *Chenopodium vulvaria* was confirmed in the area. No direct evidence of persistence may be inferred from its presence yet, because multiple independent introductions may have taken place.

The second locality of *Chenopodium vulvaria* was observed by Lazkov in the vicinity of Nookat village, Alay Range, without further information on the invasion status.

We consider this species to be alien to the country because of its weedy nature, anthropogenous characters of its habitats, and the paucity of its records (Fig. 8) which all are very recent.

#### Invasion status in Kyrgyzstan

Not documented; probably locally established (the status is inferred from the persistence of the species in the other countries of Central Asia). The observed populations are sparse and do not pose any threat to the native flora.

### Anthemis ruthenica

Bieb. 1808

urn:lsid:ipni.org:names:177582-1

#### Materials

- **Type status:** Other material. **Occurrence:** catalogNumber: 1; recordedBy: M.Pimenov, E.Kluykov, G.Lazkov; **Taxon:** family: Asteraceae; genus: Anthemis; specificEpithet: ruthenica; taxonRank: species; scientificNameAuthorship: Bieb.; **Location:** continent: Asia; country: Kyrgyzstan; stateProvince: Chüy Region; locality: Northern side of Kyrgyz Ala-Too, near Belogorka [Tosh-Bulak] Village; decimalLatitude: 42.6733; decimalLongitude: 74.2415; **Identification:** identifiedBy: G.A.Lazkov; dateIdentified: 01/01/1998; **Event:** eventDate: 06/07/1996; year: 1996; month: 6; day: 7; fieldNumber: 29; eventRemarks: alien plant; **Record Level:** collectionID: 88420; institutionCode: FRU; basisOfRecord: specimen
- **Type status:** Other material. **Occurrence:** catalogNumber: 2; recordedBy: M.Pimenov, E.Kluykov, G.Lazkov; **Taxon:** family: Asteraceae; genus: Anthemis; specificEpithet: ruthenica; taxonRank: species; scientificNameAuthorship: Bieb.; **Location:** continent: Asia; country: Kyrgyzstan; stateProvince: Chüy Region; locality: Northern side of Kyrgyz Ala-Too, near Belogorka [Tosh-Bulak] Village; decimalLatitude: 42.6733; decimalLongitude: 74.2415; **Identification:** identifiedBy: G.A.Lazkov; dateIdentified: 01/01/1998; **Event:** eventDate: 06/07/1996; year: 1996; month: 6; day: 7; fieldNumber: 29; eventRemarks: alien plant; **Record Level:** collectionID: 90565; institutionCode: LE; basisOfRecord: specimen
- **Type status:** Other material. **Occurrence:** catalogNumber: 3; recordedBy: anonym; **Taxon:** family: Asteraceae; genus: Anthemis; specificEpithet: ruthenica; taxonRank: species; scientificNameAuthorship: Bieb.; **Location:** continent: Asia; country: Kyrgyzstan; stateProvince: Chüy Region; locality: Northern side of Kyrgyz Ala-Too, near Cholok Village; decimalLatitude: 42.7493; decimalLongitude: 73.5036; **Identification:** identifiedBy: G.A.Lazkov; dateIdentified: 01/01/1998; **Event:** eventDate: 07/21/1965; year: 1965; month: 7; day: 21; fieldNumber: 1281; eventRemarks: alien plant; **Record Level:** collectionID: 88420; institutionCode: FRU; basisOfRecord: specimen
- **Type status:** Other material. **Occurrence:** catalogNumber: 4; recordedBy: Sudnitsyna; **Taxon:** family: Asteraceae; genus: Anthemis; specificEpithet: ruthenica; taxonRank: species; scientificNameAuthorship: Bieb.; **Location:** continent: Asia; country: Kazakhstan; stateProvince: Taraz Region; locality: Vicinity of Jambyl [Taraz] Town, foothills; decimalLatitude: 42.8744; decimalLongitude: 71.4376; **Identification:** identifiedBy: G.A.Lazkov; dateIdentified: 01/01/1998; **Event:** eventDate: 05/14/1970; year: 1970; month: 5; day: 14; habitat: clayey slopes, semidesert; eventRemarks: alien plant; **Record Level:** collectionID: 88420; institutionCode: FRU; basisOfRecord: specimen
- **Type status:** Other material. **Occurrence:** catalogNumber: 5; recordedBy: R.Sultanova; **Taxon:** family: Asteraceae; genus: Anthemis; specificEpithet: ruthenica; taxonRank: species; scientificNameAuthorship: Bieb.; **Location:** continent: Asia; country: Kyrgyzstan; stateProvince: Talas Region; locality: Pokrovka Village; decimalLatitude: 42.7297; decimalLongitude: 71.607; **Identification:** identifiedBy: G.A.Lazkov; dateIdentified: 01/01/1998; **Event:** eventDate: 06/02/1968; year: 1968; month: 6; day: 2; habitat: roadside; eventRemarks: alien plant; **Record Level:** collectionID: 88420; institutionCode: FRU; basisOfRecord: specimen
- **Type status:** Other material. **Occurrence:** catalogNumber: 6; recordedBy: G.Lazkov; **Taxon:** family: Asteraceae; genus: Anthemis; specificEpithet: ruthenica; taxonRank: species; scientificNameAuthorship: Bieb.; **Location:** continent: Asia; country: Kyrgyzstan; stateProvince: Chüy Region; locality: Ala-Archa River, Kashka-Suu Village; decimalLatitude: 42.6739; decimalLongitude: 74.5275; **Identification:** identifiedBy: G.A.Lazkov; dateIdentified: 01/01/1998; **Event:** eventDate: 05/01/1998; year: 1998; month: 5; eventRemarks: alien plant; **Record Level:** collectionID: 88420; institutionCode: FRU; basisOfRecord: specimen
- **Type status:** Other material. **Occurrence:** catalogNumber: 7; recordedBy: S.Sheremetova & G.Lazkov; **Taxon:** family: Asteraceae; genus: Anthemis; specificEpithet: ruthenica; taxonRank: species; scientificNameAuthorship: Bieb.; **Location:** continent: Asia; country: Kyrgyzstan; stateProvince: Chüy Region; locality: right side of Aspara River, 4 km upstream Granitogorsk Town; decimalLatitude: 42.7055; decimalLongitude: 73.458; **Identification:** identifiedBy: G.A.Lazkov; dateIdentified: 01/01/1998; **Event:** eventDate: 05/18/1990; year: 1990; month: 5; day: 18; habitat: gravelly slope along the road; eventRemarks: alien plant; **Record Level:** collectionID: 88420; institutionCode: FRU; basisOfRecord: specimen
- **Type status:** Other material. **Occurrence:** catalogNumber: 8; recordedBy: E.S.Poliakova; **Taxon:** family: Asteraceae; genus: Anthemis; specificEpithet: ruthenica; taxonRank: species; scientificNameAuthorship: Bieb.; **Location:** continent: Asia; country: Kyrgyzstan; stateProvince: Chüy Region; locality: Kara-Balta River, Taldy-Bulak; decimalLatitude: 42.6529; decimalLongitude: 73.9064; **Identification:** identifiedBy: G.A.Lazkov; dateIdentified: 01/01/1998; **Event:** eventDate: 06/17/1978; year: 1978; month: 6; day: 17; habitat: by the road; eventRemarks: alien plant; **Record Level:** collectionID: 88420; institutionCode: FRU; basisOfRecord: specimen
- **Type status:** Other material. **Occurrence:** catalogNumber: 9; recordedBy: G.Lazkov; **Taxon:** family: Asteraceae; genus: Anthemis; specificEpithet: ruthenica; taxonRank: species; scientificNameAuthorship: Bieb.; **Location:** continent: Asia; country: Kyrgyzstan; stateProvince: Chüy Region; locality: Kant Town; decimalLatitude: 42.8694; decimalLongitude: 74.8419; **Identification:** identifiedBy: G.A.Lazkov; dateIdentified: 01/01/1998; **Event:** eventDate: 01/06/2010-01/09/2010; year: 2010; eventRemarks: alien plant; **Record Level:** basisOfRecord: observation
- **Type status:** Other material. **Occurrence:** catalogNumber: 10; recordedBy: G.Lazkov; **Taxon:** family: Asteraceae; genus: Anthemis; specificEpithet: ruthenica; taxonRank: species; scientificNameAuthorship: Bieb.; **Location:** continent: Asia; country: Kyrgyzstan; stateProvince: Chüy Region; locality: Tokmok Town; decimalLatitude: 42.8105; decimalLongitude: 75.2758; **Identification:** identifiedBy: G.A.Lazkov; dateIdentified: 01/01/1998; **Event:** eventDate: 01/06/2010-01/09/2010; year: 2010; eventRemarks: alien plant; **Record Level:** basisOfRecord: observation
- **Type status:** Other material. **Occurrence:** catalogNumber: 11; recordedBy: G.Lazkov; **Taxon:** family: Asteraceae; genus: Anthemis; specificEpithet: ruthenica; taxonRank: species; scientificNameAuthorship: Bieb.; **Location:** continent: Asia; country: Kyrgyzstan; stateProvince: Chüy Region; locality: Kemin Town; decimalLatitude: 42.7595; decimalLongitude: 75.6875; **Identification:** identifiedBy: G.A.Lazkov; dateIdentified: 01/01/1998; **Event:** eventDate: 01/06/2010-01/09/2010; year: 2010; eventRemarks: alien plant; **Record Level:** basisOfRecord: observation
- **Type status:** Other material. **Occurrence:** catalogNumber: 12; recordedBy: G.Lazkov; **Taxon:** family: Asteraceae; genus: Anthemis; specificEpithet: ruthenica; taxonRank: species; scientificNameAuthorship: Bieb.; **Location:** continent: Asia; country: Kyrgyzstan; stateProvince: Chüy Region; locality: E of Kara-Balta Town, factory area (under construction); decimalLatitude: 42.798333; decimalLongitude: 73.888611; **Identification:** identifiedBy: G.A.Lazkov; dateIdentified: 22/06/2013; **Event:** eventDate: 22/06/2013; year: 2013; month: 6; day: 22; habitat: ruderal places; eventRemarks: alien plant; **Record Level:** collectionID: 88420; institutionCode: FRU; basisOfRecord: specimen

#### Native distribution and occurrence in Central Asia

*Anthemis ruthenica* is an annual or biennial species native to southeastern Europe and the Caucasus (Tzvelev 1994). There are very few published records of this alien species from Central Asia. Earlier it was known from Magtymguly Village (Kopetdagh Range, Turkmenistan), western parts of Talas and Kyrgyz Ranges in Kazakhstan, and along Shakhimardan River in Uzbekistan (Kamelin and Kovalevskaya 1993). The localities in Kazakhstan are very close to the border of Kyrgyzstan, and this proximity is confirmed by a specimen at FRU that was collected at the distance of ca 10 km (vicinity of Jambyl [Taraz] Town, foothills, clayey slopes, semidesert, 14.05.1970, *I.Sudnitsyna*). The locality in Uzbekistan is situated in the enclave surrounded by the territory of Kyrgyzstan. The presence of this species in Kyrgyzstan was therefore expected.

#### Occurrence in Kyrgyzstan

The species was first reported from Kyrgyzstan on the basis of a single recent collection from Tosh-Bulak [formerly Belogorka] village on the northern side of Kyrgyz Range (Lazkov 1999). A further revision of collections at FRU revealed a number of earlier specimens of the species collected along the foothills of the northern side of Kyrgyz Range between the border with Kazakhstan and Kara-Balta Town. The species has been most frequently collected at the country border. Besides, a single herbarium specimen was collected in the Talas Depression, also very close to the border with Kazakhstan. These records make an extension of the previously known distribution area.

The species is also known from the eastern part of the Chüy Depression. It was observed by G.Lazkov (without voucher specimens) near Kant Town, Tokmok Town, and Kemin Town of Chüy Region.

**Figure 1.**
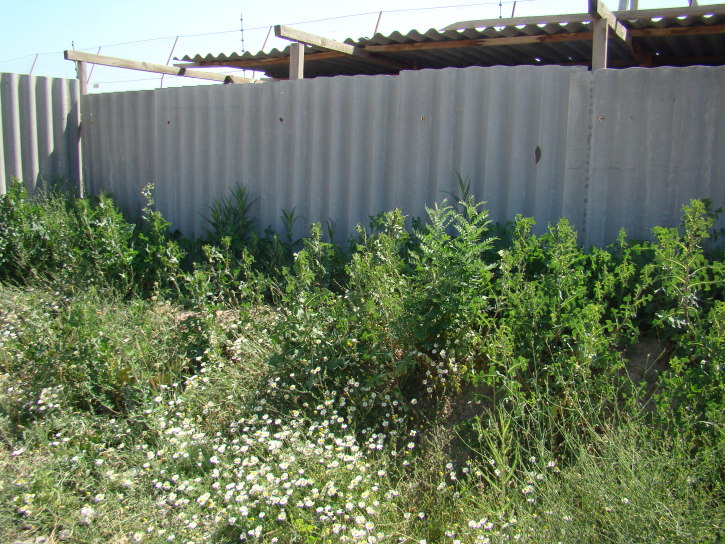
Vigorous plants of *Anthemis ruthenica* on a wasteland. Kara-Balta Town, 22.06.2013. Photo: G.Lazkov.

**Figure 2.**
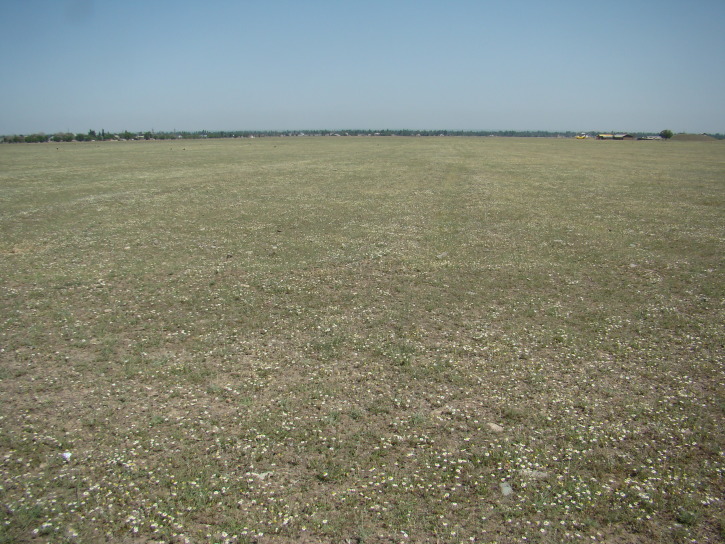
Local occurrences of *Anthemis ruthenica* may be really extensive. Kara-Balta Town, 22.06.2013. Photo: G.Lazkov.

At present, in Kyrgyzstan *Anthemis ruthenica* is widespread and locally abundant in the Chüy Depression and the western part of the Talas Depression (including foothills), completely covering the lowlands of northern Kyrgyzstan (Fig. 10). It typically occurs in wastelands, on roadsides and dry riversides. The occurrence of this species in Kyrgyzstan is not new but has been very poorly known; previously it was misidentified in collections of FRU as *Pyrethrum transiliense* (Herd.) Regel & Schmalh. The species has a clear tendency to get established in new territories and may potentially spread much further. Our latest observation comes from the vicinity of Kara-Balta Town from which the species was first recorded as late as 1978; vigorous stands were observed in the new factory area (Fig. 1). At present the species is truly abundant in disturbed lands of various kind around the town (Fig. 2).

Since most of the older records originated from the territories neighbouring with Kazakhstan, we conclude that the species was imported from that country in mid-Soviet times. The oldest record documented by a specimen collected in 1965 came indeed from Cholok-Aryk Village at the very border with Kazakhstan.

#### Invasion status in Kyrgyzstan

Established alien, naturalised (self-sustaining with established populations of a high number of individuals) in human-made and disturbed habitats at the regional scale. Spreading and invasive, locally replacing other species.

### Centaurea solstitialis

L. 1753

urn:lsid:ipni.org:names:191626-1

#### Materials

- **Type status:** Other material. **Occurrence:** catalogNumber: 13; recordedBy: A. Sennikov & G. Lazkov; **Taxon:** family: Asteraceae; genus: Centaurea; specificEpithet: solstitialis; taxonRank: species; scientificNameAuthorship: L.; **Location:** continent: Asia; country: Kyrgyzstan; stateProvince: Jalal-Abad Region; locality: Fergana Range (its SW foothills), NW of Suzak Town; verbatimElevation: 865; decimalLatitude: 40.937032; decimalLongitude: 72.889226; **Identification:** identifiedBy: G.A.Lazkov; dateIdentified: 15/08/2013; **Event:** eventDate: 15/08/2013; year: 2013; month: 8; day: 15; habitat: Red clay hills, open slopes with sparse vegetation, planted with Pistacia; fieldNumber: 280; eventRemarks: alien plant; **Record Level:** collectionID: 88420; institutionCode: FRU; basisOfRecord: specimen
- **Type status:** Other material. **Occurrence:** catalogNumber: 14; recordedBy: M.Pimenov, E.Kluykov, G.Lazkov; **Taxon:** family: Asteraceae; genus: Centaurea; specificEpithet: solstitialis; taxonRank: species; scientificNameAuthorship: L.; **Location:** continent: Asia; country: Kyrgyzstan; stateProvince: Jalal-Abad Region; locality: At-Oinok Mts.: Kürp-Say River ravine, by the mouth of the river; decimalLatitude: 41.4921; decimalLongitude: 72.3356; **Identification:** identifiedBy: G.A.Lazkov; dateIdentified: 24/06/2000; **Event:** eventDate: 24/06/2000; year: 2000; month: 6; day: 24; habitat: S-exposed slope; eventRemarks: alien plant; **Record Level:** collectionID: 88420; institutionCode: FRU; basisOfRecord: specimen
- **Type status:** Other material. **Occurrence:** catalogNumber: 15; recordedBy: M.Pimenov, E.Kluykov, G.Lazkov; **Taxon:** family: Asteraceae; genus: Centaurea; specificEpithet: solstitialis; taxonRank: species; scientificNameAuthorship: L.; **Location:** continent: Asia; country: Kyrgyzstan; stateProvince: Jalal-Abad Region; locality: At-Oinok Mts.: Kürp-Say River ravine, by the mouth of the river; decimalLatitude: 41.4921; decimalLongitude: 72.3356; **Identification:** identifiedBy: G.A.Lazkov; dateIdentified: 24/06/2000; **Event:** eventDate: 24/06/2000; year: 2000; month: 6; day: 24; habitat: S-exposed slope; eventRemarks: alien plant; **Record Level:** collectionID: 90565; institutionCode: LE; basisOfRecord: specimen

#### Native distribution and occurrence in Central Asia

In Central Asia this species is native to Turkmenistan, Tajikistan and (with a limited distribution) Uzbekistan. It occurs on dry gravelly and clayey slopes in lowlands and foothills, and is frequently found on arable lands and wastelands, in orchards, and along roadsides and artificial brooks (Makhmedov 1993).

#### Occurrence in Kyrgyzstan

**Figure 3.**
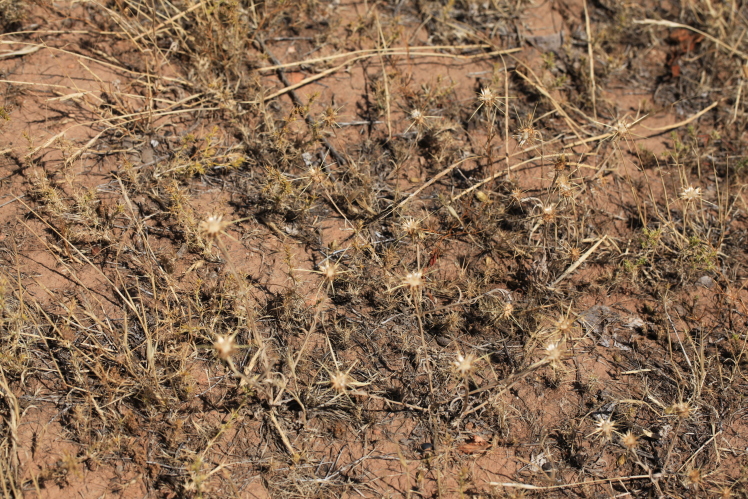
Plants of *Centaurea solstitialis* at Topurak-Bel Pass, 15.08.2013. Photo: A.Sennikov.

**Figure 4.**
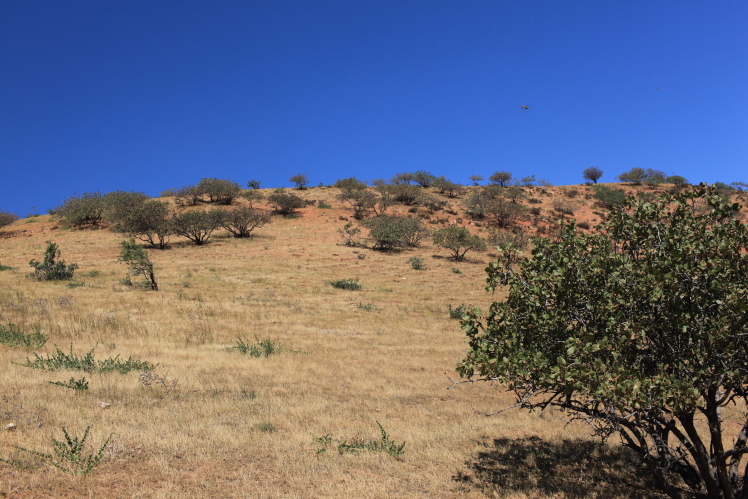
Habitat of *Centaurea solstitialis* at Topurak-Bel Pass, 15.08.2013. Photo: A.Sennikov.

In Kyrgyzstan the only known population of this species, first recorded in 2000, had persisted in At-Oinok Mts. for a few years (Lazkov 2001), but the plants have no longer been found recently (Lazkov, pers. obs.). This population was situated in a ravine that is known for, among other activities, historical cultivation of fruit crops that still survived there as old big trees of *Morus alba* L. and *Juglans regia* L., and a single thick-trunked liana of *Vitis vinifera* L. In 2013 we observed another population of a few dozens of individuals (Fig. 3) in the southwestern foothills of the Fergana Range, near Topurak-Bel Pass, where it occurred in a sparse vegetation of the hemisavannah type on the hills of red clay planted with pistachio trees close to an isolated homestead (Fig. 4). The hills were almost completely turned into a pistachio garden in the Soviet times; the trees are still in good condition and bear a plenty of fruits. No other alien plants were observed in the site.

From the sporadic and recent character of records (Fig. 8) we infer that the species is alien to the country, most likely transported with agricultural activities in the late Soviet times from the other countries of Central Asia.

#### Invasion status in Kyrgyzstan

Judging from the population size at the Topurak-Bel and its good seed set, this hardy annual is locally established in Kyrgyzstan. No spread is observed from the locality, and its future is uncertain.

### Crambe orientalis

L. 1753

urn:lsid:ipni.org:names:281660-1

Crambe amabilis Butkov & Majlun. Synonym.

#### Materials

- **Type status:** Other material. **Occurrence:** catalogNumber: 17; recordedBy: A. Sennikov & G. Lazkov; **Taxon:** family: Brassicaceae; genus: Crambe; specificEpithet: orientalis; taxonRank: species; scientificNameAuthorship: L.; **Location:** continent: Asia; country: Kyrgyzstan; stateProvince: Talas Region; locality: Maimak railway station, W of the station; decimalLatitude: 42.6762; decimalLongitude: 71.2116; **Identification:** identifiedBy: G.A.Lazkov; dateIdentified: 08/07/2013; **Event:** eventDate: 08/07/2013; year: 2013; month: 8; day: 7; habitat: railway embankment and adjacent mountain slopes; eventRemarks: alien plant; **Record Level:** basisOfRecord: observation
- **Type status:** Other material. **Occurrence:** catalogNumber: 18; recordedBy: G.Lazkov; **Taxon:** family: Brassicaceae; genus: Crambe; specificEpithet: orientalis; taxonRank: species; scientificNameAuthorship: L.; **Location:** continent: Asia; country: Kyrgyzstan; stateProvince: Chüy Region; locality: 4 km NW of Ysyk-Ata Resort; verbatimElevation: 1000; decimalLatitude: 42.6376; decimalLongitude: 74.9348; **Identification:** identifiedBy: G.A.Lazkov; dateIdentified: 05/23/2006; **Event:** eventDate: 05/23/2006; year: 2006; month: 5; day: 23; habitat: allotment garden; eventRemarks: alien plant; **Record Level:** collectionID: 88420; institutionCode: FRU; basisOfRecord: specimen
- **Type status:** Other material. **Occurrence:** catalogNumber: 19; recordedBy: G.Lazkov; **Taxon:** family: Brassicaceae; genus: Crambe; specificEpithet: orientalis; taxonRank: species; scientificNameAuthorship: L.; **Location:** continent: Asia; country: Kyrgyzstan; stateProvince: Chüy Region; locality: Chüy Depression, railway crossing by Petrovka Village; decimalLatitude: 42.8218; decimalLongitude: 74.0508; **Identification:** identifiedBy: G.A.Lazkov; dateIdentified: 08/28/2006; **Event:** eventDate: 08/28/2006; year: 2006; month: 8; day: 28; eventRemarks: alien plant; **Record Level:** collectionID: 88420; institutionCode: FRU; basisOfRecord: specimen
- **Type status:** Other material. **Occurrence:** catalogNumber: 20; recordedBy: G.Lazkov; **Taxon:** family: Brassicaceae; genus: Crambe; specificEpithet: orientalis; taxonRank: species; scientificNameAuthorship: L.; **Location:** continent: Asia; country: Kyrgyzstan; stateProvince: Chüy Region; locality: Bishkek City, by railway bridge across Manas Avenue; decimalLatitude: 42.8651; decimalLongitude: 74.587; **Identification:** identifiedBy: G.A.Lazkov; dateIdentified: 08/28/2006; **Event:** eventDate: 08/28/2006; year: 2006; month: 8; day: 28; eventRemarks: alien plant; **Record Level:** collectionID: 88420; institutionCode: FRU; basisOfRecord: specimen
- **Type status:** Other material. **Occurrence:** catalogNumber: 21; recordedBy: G.Lazkov; **Taxon:** family: Brassicaceae; genus: Crambe; specificEpithet: orientalis; taxonRank: species; scientificNameAuthorship: L.; **Location:** continent: Asia; country: Kyrgyzstan; stateProvince: Chüy Region; locality: Besh-Küngöy; decimalLatitude: 42.775; decimalLongitude: 74.655; **Identification:** identifiedBy: G.A.Lazkov; dateIdentified: 04/28/2006; **Event:** eventDate: 04/28/2006; year: 2006; month: 4; day: 28; habitat: foothills; eventRemarks: alien plant; **Record Level:** collectionID: 88420; institutionCode: FRU; basisOfRecord: specimen

#### Native distribution and occurrence in Central Asia

This perennial species is native to Western Asia, ranging from Turkey to the *Flora Iranica* area (Hedge 1968), including Turkmenistan (Hedge 1968; Nikitin and Geldykhanov 1988).

The first record of the alien *Crambe orientalis* in Western Tian-Shan is dated 1922 when a specimen was collected from the foothills between Shymkent Town (Kazakhstan) and Angren River (Uzbekistan). The species occurred abundantly on grain fields and abandoned lands in this limited area, from where it was erroneously described and subsequently accepted as the local endemic *Crambe amabilis* Butkov & Majlun (Kovalevskaya 1974). Most probably it was transported to the place with cultivated plants (Botschantzev 1977).

#### Occurrence in Kyrgyzstan

**Figure 5.**
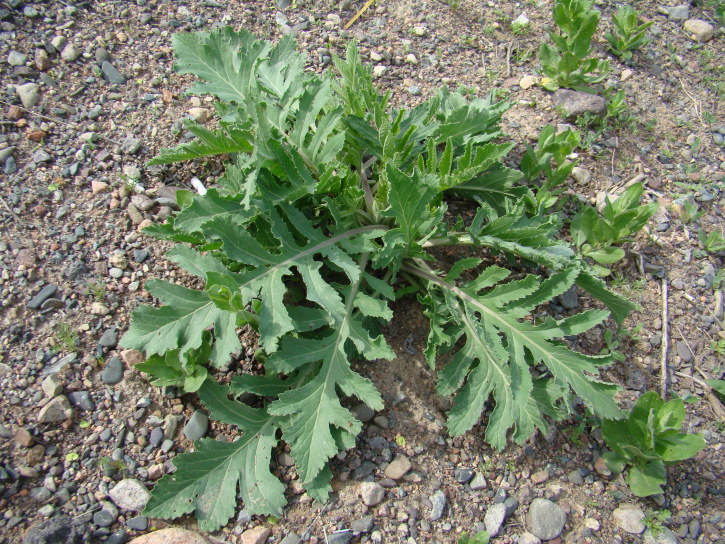
A young plant of *Crambe orientalis*. Chüy Depression, 15.04.2013. Photo: G.Lazkov.

In Kyrgystan *Crambe orientalis* was first recorded from the Chüy Depression in 2006 (Lazkov and Redina 2007). The present new record (that is a novelty to the Talas Depression) fills the gap in the secondary distribution area (Fig. 8). As in the Chüy Depression, the species was observed established along the railway embankments which are its major channel of dispersal, but single plants (Fig. 5) were noticed outside the railway area on the neighbouring mountain slopes.

Most likely the plants arrived from Kazakhstan where *Crambe orientalis* was commonly found in the southern territories (Lazkov and Redina 2007). In northern Kyrgyzstan the species was also observed as a garden weed (Lazkov and Redina 2007), demonstrating multiple sources and means of dispersal and possibly arrival to the country.

#### Invasion status in Kyrgyzstan

Established alien, spreading further along railways and with cultivated plants. Potentially invasive (as demonstrated by its naturalization in Kazakhstan and Uzbekistan), although at present almost entirely confined to human-made habitats.

### Hirschfeldia incana

(L.) Lagr.-Foss. 1847

urn:lsid:ipni.org:names:285350-1

Erucastrum incanum (L.) W.D.J.Koch. Synonym.

#### Materials

- **Type status:** Other material. **Occurrence:** catalogNumber: 22; recordedBy: G.Lazkov; **Taxon:** family: Brassicaceae; genus: Hirschfeldia; specificEpithet: incana; taxonRank: species; scientificNameAuthorship: (L.) Lagr.-Foss.; **Location:** continent: Asia; country: Kyrgyzstan; stateProvince: Jalal-Abad Region; locality: Fergana Range: Kara-Ünkür River basin, Kyr-Koo Village; decimalLatitude: 41.1876; decimalLongitude: 72.911; **Identification:** identifiedBy: G.A.Lazkov; dateIdentified: 06/03/2013; **Event:** eventDate: 06/03/2013; year: 2013; month: 6; day: 3; habitat: among shrubs; eventRemarks: alien plant; **Record Level:** collectionID: 88420; institutionCode: FRU; basisOfRecord: specimen

#### Native distribution and occurrence in Central Asia

*Hirschfeldia incana* is a Mediterranean species that has never been recorded from Central Asia as a whole. The closest approach of its native distribution area is in Iran (Hedge 1968).

#### Occurrence in Kyrgyzstan

New country record, and the first record in Central Asia. A single plant was noticed and collected in 2013 among shrubs in Kyr-Koo Village on the western side of Fergana Range (Fig. 9), but the area has not been specially explored for the species.

#### Invasion status in Kyrgyzstan

Most likely this is a casual, ephemeral introduction, caused by long-distance dispersal.

### Salvia aethiopis

L. 1753

urn:lsid:ipni.org:names:455581-1

#### Materials

- **Type status:** Other material. **Occurrence:** catalogNumber: 27; recordedBy: D.Milko; **Taxon:** family: Lamiaceae; genus: Salvia; specificEpithet: aethiopis; taxonRank: species; scientificNameAuthorship: L.; **Location:** continent: Asia; country: Kyrgyzstan; stateProvince: Ysyk-Köl Region; locality: 1.5 km N of Kürmöntü Vilage; decimalLatitude: 42.8161; decimalLongitude: 78.2407; **Identification:** identifiedBy: G.A.Lazkov; dateIdentified: 01/01/2011; **Event:** eventDate: 01/01/2011; year: 2011; eventRemarks: alien plant; **Record Level:** basisOfRecord: observation
- **Type status:** Other material. **Occurrence:** catalogNumber: 28; recordedBy: G.Lazkov & U.Neveraev; **Taxon:** family: Lamiaceae; genus: Salvia; specificEpithet: aethiopis; taxonRank: species; scientificNameAuthorship: L.; **Location:** continent: Asia; country: Kyrgyzstan; stateProvince: Batken Region; locality: Orozbekovo Village; decimalLatitude: 40.062; decimalLongitude: 71.666; **Identification:** identifiedBy: G.A.Lazkov; dateIdentified: 06/10/2012; **Event:** eventDate: 06/10/2012; year: 2012; month: 6; day: 10; eventRemarks: alien plant; **Record Level:** collectionID: 88420; institutionCode: FRU; basisOfRecord: specimen
- **Type status:** Other material. **Occurrence:** catalogNumber: 29; recordedBy: G.Lazkov; **Taxon:** family: Lamiaceae; genus: Salvia; specificEpithet: aethiopis; taxonRank: species; scientificNameAuthorship: L.; **Location:** continent: Asia; country: Kyrgyzstan; stateProvince: Chüy Region; locality: Kemin Village; decimalLatitude: 42.78; decimalLongitude: 75.71; **Identification:** identifiedBy: G.A.Lazkov; dateIdentified: 07/20/2012; **Event:** eventDate: 07/20/2012; year: 2012; month: 7; day: 20; eventRemarks: alien plant; **Record Level:** collectionID: 88420; institutionCode: FRU; basisOfRecord: specimen
- **Type status:** Other material. **Occurrence:** catalogNumber: 30; recordedBy: A. Sennikov & G. Lazkov; **Taxon:** family: Lamiaceae; genus: Salvia; specificEpithet: aethiopis; taxonRank: species; scientificNameAuthorship: L.; **Location:** continent: Asia; country: Kyrgyzstan; stateProvince: Talas Region; locality: Karacha-Too Mts. (E part of Kara-Tau); verbatimElevation: 865; decimalLatitude: 42.675043; decimalLongitude: 71.192088; **Identification:** identifiedBy: G.A.Lazkov; dateIdentified: 08/07/2013; **Event:** eventDate: 08/07/2013; year: 2013; month: 8; day: 7; habitat: lowermost part of slopes near the railway; fieldNumber: 180; eventRemarks: alien plant; **Record Level:** collectionID: 88420; institutionCode: FRU; basisOfRecord: specimen
- **Type status:** Other material. **Occurrence:** catalogNumber: 31; recordedBy: A. Sennikov & G. Lazkov; **Taxon:** family: Lamiaceae; genus: Salvia; specificEpithet: aethiopis; taxonRank: species; scientificNameAuthorship: L.; **Location:** continent: Asia; country: Kyrgyzstan; stateProvince: Talas Region; locality: Talas Range (N side), Kolbars; verbatimElevation: 1580; decimalLatitude: 42.445237; decimalLongitude: 71.049844; **Identification:** identifiedBy: G.A.Lazkov; dateIdentified: 08/08/2013; **Event:** eventDate: 08/08/2013; year: 2013; month: 8; day: 8; habitat: open semidesert slopes; fieldNumber: 196; eventRemarks: alien plant; **Record Level:** collectionID: 88420; institutionCode: FRU; basisOfRecord: specimen

#### Native distribution and occurrence in Central Asia

In Central Asia the distribution area of *Salvia aethiopis* has three isolated fragments (Makhmedov 1984, Makhmedov 1987), of which the mountainous occurrence in Turkmenistan (Borisova 1954) and southern Uzbekistan (Vvedensky 1961) may be considered native. Two isolated localities nearby Mikhailovka and Lugovoe Villages in Kazakhstan (Makhmedov 1984, Makhmedov 1987), which are very close to the border with Kyrgyszstan, are situated on the lowlands north of Kyrgyz Range (not in the foothills of Kyrgyz Range as stated in Makhmedov 1984) and obviously constitute an alien occurrence.

#### Occurrence in Kyrgyzstan

This conspicuous species had not been recorded from Kyrgyzstan until Lazkov et al. 2012 reported its presence on the southern side of Küngöy Ala-Too, the northern side of Alay Range and in the Chüy Depression, where it was discovered in 2011–2012. Our records extend the known occurrence of this species to the Talas Depression (Karacha-Too Mountains) and the neighbouring side of Talas Range (Kök-Say River valley), very close to the border with Kazakhstan (Fig. 9).

Its arrival from Kazakhstan may have been by the means of wind transportation because the dried plants are easy to get detached from the ground, forming a tumbleweed. The means of long-distance dispersal to the other territories are uncertain.

#### Invasion status in Kyrgyzstan

Several plants were observed in every locality, meaning that the species is most likely established in the country. Its further spread, especially in lowlands, is expected. The observed populations are usually sparse and pose no obvious threat to the native vegetation.

### Glaucium corniculatum

(L.) Rudolph

urn:lsid:ipni.org:names:161608-3

#### Materials

- **Type status:** Other material. **Occurrence:** catalogNumber: 25; recordedBy: Manual of vascular plants of Central Asia; **Taxon:** family: Papaveraceae; genus: Glaucium; specificEpithet: corniculatum; taxonRank: species; scientificNameAuthorship: (L.) Curt.; **Location:** continent: Asia; country: Kyrgyzstan; stateProvince: Chüy Region; locality: Kant Town, as a weed on experimental fields; decimalLatitude: 42.945; decimalLongitude: 74.828; **Identification:** identifiedBy: V.K.Pazij; dateIdentified: 01/01/1974; **Event:** eventDate: 01/01/1974-31/12/1974; year: 1974; habitat: experimental field, weed; eventRemarks: alien plant; **Record Level:** basisOfRecord: literature
- **Type status:** Other material. **Occurrence:** catalogNumber: 26; recordedBy: G.Lazkov; **Taxon:** family: Papaveraceae; genus: Glaucium; specificEpithet: corniculatum; taxonRank: species; scientificNameAuthorship: (L.) Curt.; **Location:** continent: Asia; country: Kyrgyzstan; stateProvince: Chüy Region; locality: E of Kara-Balta Town, factory area (under construction); decimalLatitude: 42.798333; decimalLongitude: 73.888611; **Identification:** identifiedBy: G.A.Lazkov; dateIdentified: 22/06/2013; **Event:** eventDate: 22/06/2013; year: 2013; month: 6; day: 22; habitat: ruderal places; eventRemarks: alien plant; **Record Level:** collectionID: 88420; institutionCode: FRU; basisOfRecord: specimen

#### Native distribution and occurrence in Central Asia

In Central Asia this species is native to Kopetdagh, Turkmenistan (Nikitin and Geldykhanov 1988). Outside its native distribution area, it has been sporadically found in Turkmenistan (ruderal in oases), Uzbekistan (Tashkent City, roadsides; Fergana City, ruderal), and Kyrgyzstan (Pazij 1974).

#### Occurrence in Kyrgyzstan

**Figure 6.**
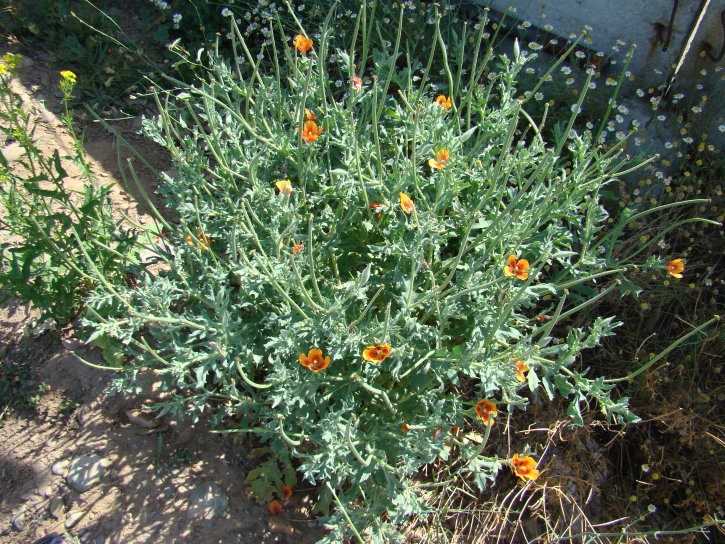
The plants of *Glaucium corniculatum* are very vigorous on waste ground. Kara-Balta Town, 22.06.2013. Photo: G.Lazkov.

In Kyrgyzstan, this species had only been recorded as ruderal on experiental fields in the Chüy Depression (Pazij 1974) on the basis of collections kept at TASH. The second record in Kyrgyzstan was made in 2013 in the factory area east of Kara-Balta Town, lower part of N side of Kyrgyz Range (Fig. 9), together with *Chenopodium vulvaria* and *Anthemis ruthenica* reported here. A few flowering individuals (Fig. 6) were noticed.

#### Invasion status in Kyrgyzstan

Because of the low number of individuals and the ephemerous nature of the previous record, the occurrence at Kara-Balta looks casual. The species has not yet been established in Kyrgyzstan.

### Arrhenatherum elatius

J.Presl & C.Presl 1819

urn:lsid:ipni.org:names:140632-3

#### Materials

- **Type status:** Other material. **Occurrence:** catalogNumber: 32; recordedBy: student excursion of the Department of botany and plant physiology, Kyrgyz National University; **Taxon:** family: Poaceae; genus: Arrhenatherum; specificEpithet: elatius; taxonRank: species; scientificNameAuthorship: (L.) J.Presl & C.Presl; **Location:** continent: Asia; country: Kyrgyzstan; stateProvince: Ysyk-Köl Region; locality: Vicinity of Bosteri Village; decimalLatitude: 42.6464; decimalLongitude: 77.1656; **Identification:** identifiedBy: G.A.Lazkov; dateIdentified: 08/15/2013; **Event:** eventDate: 01/06/2013-31/06/2013; year: 2013; month: 6; eventRemarks: alien plant; **Record Level:** collectionID: 88420; institutionCode: FRU; basisOfRecord: specimen
- **Type status:** Other material. **Occurrence:** catalogNumber: 33; recordedBy: A. Sennikov & G. Lazkov; **Taxon:** family: Poaceae; genus: Arrhenatherum; specificEpithet: elatius; taxonRank: species; scientificNameAuthorship: (L.) J.Presl & C.Presl; **Location:** continent: Asia; country: Kyrgyzstan; stateProvince: Talas Region; locality: Talas Valley, Kök-Say Village; verbatimElevation: 1220; decimalLatitude: 42.510106; decimalLongitude: 71.117674; **Identification:** identifiedBy: A.N.Sennikov; dateIdentified: 08/08/2013; **Event:** eventDate: 08/08/2013; year: 2013; month: 8; day: 8; habitat: Remnants of old cultivation along an artificial brook, dispersed and established; fieldNumber: 195; eventRemarks: alien plant; **Record Level:** collectionID: 88420; institutionCode: FRU; basisOfRecord: specimen

#### Native distribution and occurrence in Central Asia

This economically important species is very commonly cultivated in Europe as forage and ornamental plant, often escaping and getting established (Tzvelev 1976). Its native distribution area lies in Europe, the Mediterranean and Western Asia (Tzvelev 1976). In the mountainous Central Asia, in addition to the native occurrence in Turkmenistan (Rozhevits 1932, Nikitin and Geldykhanov 1988), it was locally cultivated on experimental fields but had never been commonly introduced (Sidorenko 1957). In Kazakhstan *Arrhenatherum elatius* was cultivated for artificial grasslands and lawns, and characterized as easy to run wild (Gamayunova 1956).

#### Occurrence in Kyrgyzstan

This species has never been reported from Kyrgyzstan. We observed *Arrhenatherum elatius* growing as self-sawn relics of abandoned cultivation in the private garden in Kök-Say Village (southwestern part of the Talas Depression), originally planted for forage and now spreading along artificial brooks outside the village. Another record (Fig. 8) comes from a student excursion to the northern side of the Ysyk-Köl Lake, where the species was cultivated as ornamental plant in the resort area.

#### Invasion status in Kyrgyzstan

The species may be considered locally established at Kök-Say because of its persistence and spread from the place of original cultivation. In the observed place the species does not show obvious threats to the native vegetation. The invasion status of the other locality is not ascertained.

### Tragus racemosus

(L.) All. 1785

urn:lsid:ipni.org:names:1084305-2

#### Materials

- **Type status:** Other material. **Occurrence:** catalogNumber: 34; recordedBy: G.Lazkov; **Taxon:** family: Poaceae; genus: Tragus; specificEpithet: racemosus; taxonRank: species; scientificNameAuthorship: (L.) Desf.; **Location:** continent: Asia; country: Kyrgyzstan; stateProvince: Chüy Region; locality: Chüy Depression, Tokmok Town; decimalLatitude: 42.857; decimalLongitude: 75.29; **Identification:** identifiedBy: G.A.Lazkov; dateIdentified: 06/18/2013; **Event:** eventDate: 06/18/2013; year: 2013; month: 6; day: 18; habitat: side of by-pass highway; eventRemarks: alien plant; **Record Level:** collectionID: 88420; institutionCode: FRU; basisOfRecord: specimen

#### Native distribution and occurrence in Central Asia

*Tragus racemosus* is mainly zoochorous because its diasporas may easily get attached to the cattle’s wool (Tzvelev 1976). This pasture weed, widely distributed in the Mediterranean, southeastern Europe, Asia Minor and Iran, has not been recorded in Central Asia outside its native area in Turkmenistan and doubtfully native presence in the westernmost Kazakhstan (Tzvelev 1976).

#### Occurrence in Kyrgyzstan

**Figure 7.**
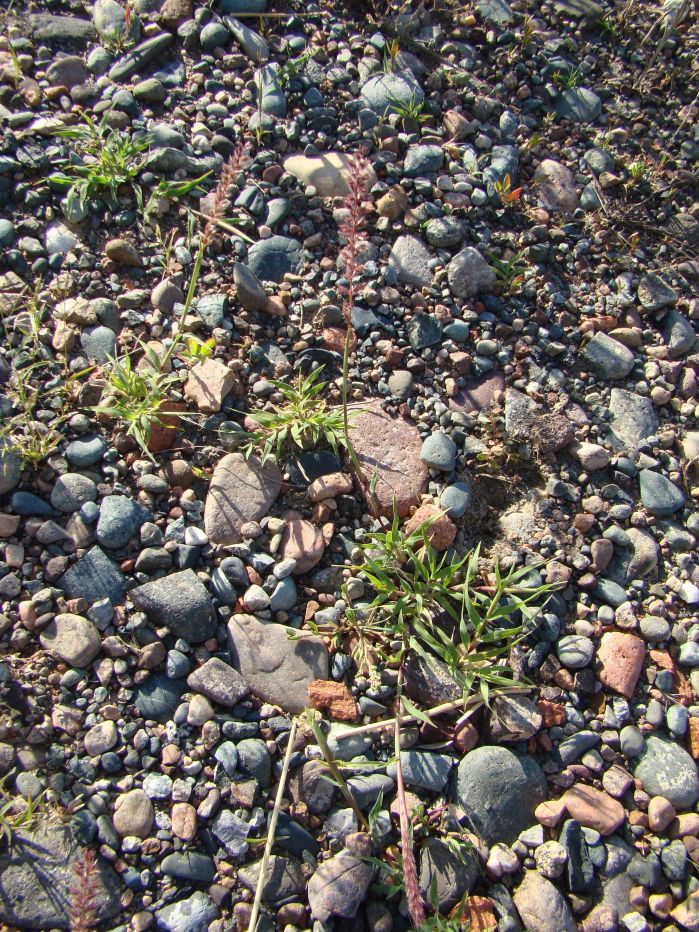
A sparse colony of *Tragus racemosus* on a roadside nearby Tokmok Town. 18.06.2013. Photo: G.Lazkov.

**Figure 8.**
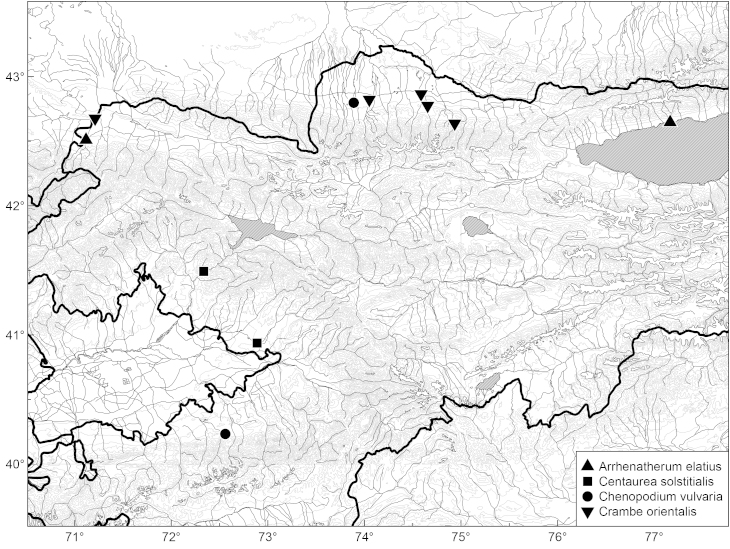
Distribution of *Arrhenatherum elatius*, *Centaurea solstitialis*, *Chenopodium vulvaria* and *Crambe orientalis* in Kyrgyzstan.

**Figure 9.**
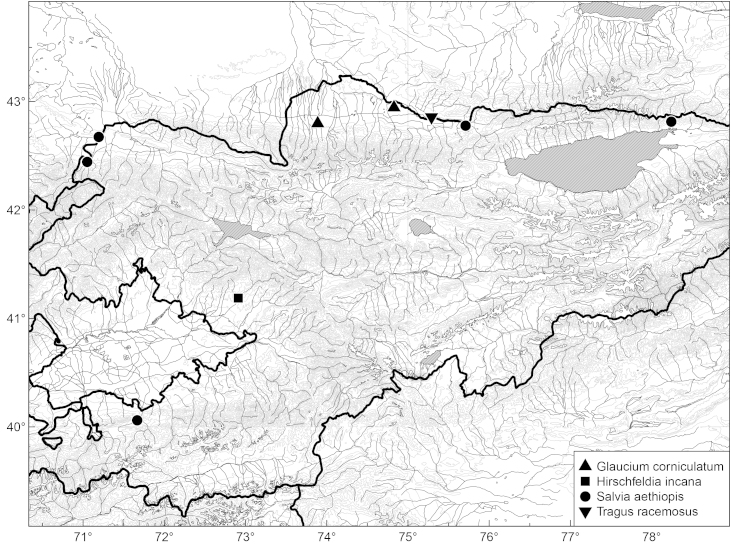
Distribution of *Glaucium corniculatum*, *Hirschfeldia incana*, *Salvia aethiopis* and *Tragus racemosus* in Kyrgyzstan.

**Figure 10.**
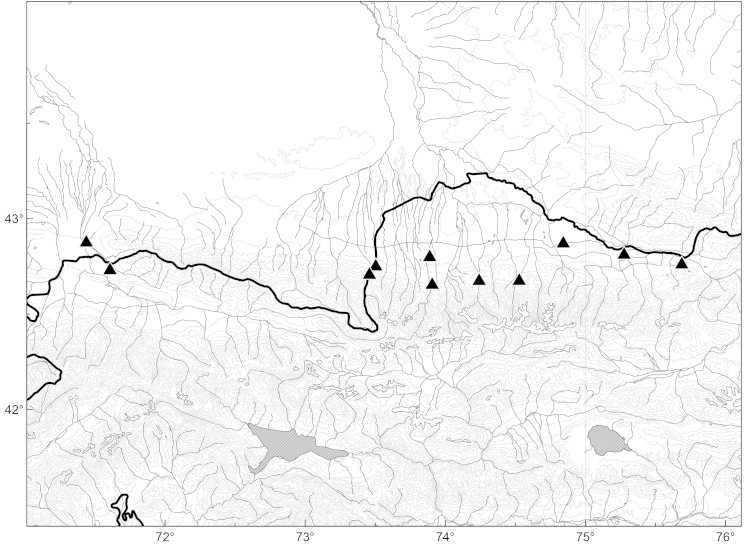
Distribution of *Anthemis ruthenica* (triangles) in Kyrgyzstan.

The present record is the first in Kyrgyzstan and also the first alien occurrence recorded with certainty in Central Asia. A few mature plants (Fig. 7) were observed along the roadside close to Tokmok Town, eastern part of the Chüy Depression (Fig. 9). Diasporas of *Tragus racemosus* may have been transported to the place with cattle because roadsides of any kind in the proximity of villages and towns are commonly used as pathways for cattle.

#### Invasion status in Kyrgyzstan

The invasion status of *Tragus racemosus* in Kyrgyzstan is not ascertained yet but its only known occurrence is likely ephemerous. For this reason the species is provisionally assessed as a casual alien in the country.

## Discussion

All the species reported here were found in human-made or disturbed habitats in a close proximity to or within inhabited places or industrial areas. These records do not show a clear pattern of geographical distribution, indicating that there may be many different means and vectors of invasion, yet to be analysed in the future.

In total, only 74 species of vascular plants are currently known as aliens in Kyrgyzstan, either casual or established. We expect that a further exploration of the flora of Kyrgyzstan will bring much more novelties in non-native plants, because the alien flora in its entirety has never been subjected to a separate study in this country.

## Supplementary Material

Supplementary material 1 Darwin Core Archive file of new records in alien vascular plants of Kyrgyzstan Data type: Occurrences
Brief description: The dataset for the present article, Darwin Core formatted in a single file.
File: oo_5444.xlsx Lazkov, G.A & Sennikov, A.N.

XML Treatment for Chenopodium vulvaria

XML Treatment for Anthemis ruthenica

XML Treatment for Centaurea solstitialis

XML Treatment for Crambe orientalis

XML Treatment for Hirschfeldia incana

XML Treatment for Salvia aethiopis

XML Treatment for Glaucium corniculatum

XML Treatment for Arrhenatherum elatius

XML Treatment for Tragus racemosus
